# Effect of an aged wearing suit on nursing student’s knowledge and attitude

**DOI:** 10.1186/s12912-021-00668-2

**Published:** 2021-08-16

**Authors:** Zahra Mandegari Bamakan, Khadijeh Nasiriani, Farzan Madadizadeh, Fatemeh Keshmiri

**Affiliations:** 1 Department of Nursing, International Campus, Shahid Sadoughi University of Medical Sciences, Yazd, Iran; 2 Department of Nursing, School of Nursing & Midwifery, Mother and Newborn Health Research Center, Research Centre for Nursing and Midwifery Care, Shahid Sadoughi University of Medical Sciences, Yazd, Iran; 3 Center for healthcare Data modeling, Departments of biostatistics and Epidemiology, School of public health, Shahid Sadoughi University of Medical Sciences, Yazd, Iran; 4 Medical Education Department, Educational Developmental Center, School of Public Health, Shahid Sadoughi University of Medical Sciences, Yazd, Iran

**Keywords:** Aging, Education, Geriatric, Knowledge, Attitude, Nursing Student, Simulation

## Abstract

**Background:**

The knowledge and attitude of health care providers are important and influential factors in providing care services to the elderly and need to be considered during the training course. Simulation in geriatric nursing education can be an opportunity for learners to experience the restrictions of the elderly. The present study was conducted to determine the effect of training through simulation on the attitude and knowledge of nursing students in elderly care.

**Methods:**

This study was quasi-experimental with two experimental and control groups of pre and post-test, which was conducted on 70 nursing students of the 5th semester (two groups of 35 people). For the experimental group, the elderly simulation suit was worn for two hours, which was designed by the researcher and created sensory, physical, and motor restrictions similar to the elderly for students. Before and after the study, Kogan’s attitudes toward older people scale and Palmore’s “facts on aging quiz” were completed by students. The data were analyzed using an independent t-test and paired t-test using SPSS version 16 software.

**Results:**

The mean scores of students’ knowledge in the experimental and control groups had no significant difference at the beginning of the study (*p* < 0.05). But the mean scores of knowledge in the experimental group before and after the intervention was (9.2 ± 2.6) and (15.3 ± 3.5), respectively, and in the control group before and after the intervention was (10.4 ± 2.9) and (11.3 ± 2.6), respectively, which had a statistically significant difference (*p* = 0.0001). The mean scores of students’ attitudes in the experimental and control groups had no significant difference at the beginning of the study (*p* < 0.05). The mean scores of attitude in the experimental group before and after the intervention was (114.69 ± 8.4) and (157.31 ± 10.7), respectively and in the control group before and after the intervention was (113.34 ± 13.6) and (108.5 ± 16.6), respectively, which was significantly different (*p* = 0.0001).

**Conclusions:**

Based on the findings, the experience of aging restrictions through simulation has improved the knowledge and attitude of nursing students towards the elderly. Nursing education requires the growth of attitudinal skills, individuals’ beliefs, and creating empathy among them, so creating simulation opportunities can assist nursing students in the educational processes.

## Background

Aging is a natural phenomenon involving biological, cognitive, and environmental components [1]. According to forecasts, the number of the elderly will reach about 1 billion and 200 million in 2025, which indicates the doubling of the number of elderly over the next 25 years [2]. The increase in life span is associated with an increase in the number of acute and chronic problems and diseases, leading to specific issues and requirement for health services [3]. Therefore, the focus on training healthcare workers should comply with the demand for population change and systems deficiency [4]. However, despite the increasing number of elderly in the world, the growth of aging specialists seems to be much slower than the rate of aging in the world [5]. One of the important groups that should be prepared for elderly care is nurses [6].

Elderly care is an important part of the professional duties of a nurse and helping the elderly in compliance with their physical, mental and social weaknesses is one of the duties of nurses [7]. Therefore, nurses need to be prepared to respond to the elderly needs [6]. But the training programs seem not to have been planned to provide preparation among novice nurses to care for the elderly needs. The results of studies have shown that novice nurses have problems with proper understanding and attitudes towards the elderly and do not understand the challenges related to aging [8, 9]. Understanding the issues and problems of the elderly can be difficult for healthcare workers, especially students [10]because they have not yet experienced an aging or the unpleasant experience of illness and disability that can cause an increase in the understanding of the elderly [11, 12]. Therefore, the existence of knowledge and attitude of the health care providers to the elderly is an important and influential factor in providing care services to the elderly [13]. Nursing students’ attitudes as nursing practitioner candidates play an essential role in their professionalism as nurses [9].

Although the attitude change is difficult in the education process, it is one of the most important aspects of nursing care that can play an important role in providing nursing services [13]. Achieving the necessary qualifications in this field is influenced by effective factors such as motivation, supervision, and access to appropriate education [8]. One of the proposed methods is to run simulation programs [14].Simulation attempts to display some behavioral aspects of a physical or abstract system by the behavior of another system [15]. The clinical simulation includes a wide range of methods and techniques that allow students to practice clinical skills before using them in relation with real patients [16]. The main purpose of using simulation in health care is to prepare students to face real situations that increase help-seeker safety, reduce errors and improve nurses’ clinical judgments [17, 18].

Simulation leads to an attitude change in the learners, readiness to learn new roles, helping learners to understand the professional role, playing an effective role on the learners, increasing the motivation and interest in the learner, and creating critical thinking processes in the learners [19]. The striking point is that this method should be as similar to the real environment as possible so that what the practitioners learn can be transferred to the real environment [20]. Simulating the restrictions of aging can help students to acquire the facts on aging [13] and be important in improving the knowledge of elderly care and understanding issues and problems and providing appropriate care [7]. Besides, students can experience changing appropriate functions with aging such as impairments and auditory, visual, and sedentary disorders, which are useful in understanding the feelings and experiences of the elderly [7, 13, 15].

Despite some studies conducted on the effect of simulation training in nursing, there is little empirical evidence of how this method is used, and further research is required. Especially in the field of elderly care training, which is increasingly needed, the necessity of research in this area becomes more evident. This study aimed to determine the effect of simulated-based training on the attitude and knowledge of nursing students regarding nursing care of the elderly.

## Methods

The present study is a developmental research, applied, quasi-experimental research with the experimental and control groups of pre and post-test type which has been conducted in three phases of designing, implementation, and evaluation. In the design phase, according to the educational requirements of nursing students in the field of elderly care, a simulated suit was selected and the necessary preparations were provided for the study. The participants were 70 nursing students of the 5th semester of the school of nursing and midwifery of Shahid Sadoughi university of Yazd, Iran, who were included in the study based on the inclusion and exclusion criteria. Then, class A was selected as the experimental group and class B was selected as the control group in the form of a lottery between the two classes of the same semester. The reason for the lack of randomization of both classes was to reduce bias and create closer contact of participants, also to avoid orientation in the research results. The exclusion criteria were determined to have a diploma in nursing, home care for the elderly, students on education leave and hospitality from another university, and dropping criteria for students’ non-participation in the program and incomplete completion of questionnaires were determined.

The sample size is based on the study of Haj Bagheri et al., which was conducted with the subject of the effect of the aging program on an aging nursing student’s attitude [21]. The following information was extracted: After the intervention, the mean (standard deviation) of the attitude score in the control group was equal to 125.37 (8.22) and the mean (standard deviation) of the attitude score in the intervention group was equal to (8.96) 119.62 that by considering the significance level of 5 % and the power of a test of 80 %, the sample size of 35 participants was estimated.

In the implementation phase, to conduct the study, the informed written consent was obtained from the students by explaining the research objectives to participate in the study. For the intervention group, students wore suits designed to simulate elderly restrictions. The specifications of the suit were as follows: using glasses with yellow talc to create visual restrictions, using prefabricated knee supporter to restrict the movement of the lower limbs and prefabricated elbow supporter to restrict the movement of the upper limbs, tying bags containing sand to wrist and legs in the direction of atrophy, using a designed plaster cast, and a spine function restriction belt .the aging, muscle hypertrophy were created and by gluing the fingers joints, the movement of the finger joints was limited. The suit was worn in the clinical skills laboratory with a researcher’s help. The students were asked to do the usual daily activities of the elderly. The students did to take steps such as opening a heavy iron door and entering the campus, walking down the stairs, and walking in the courtyard. After walking in the courtyard, return to the clinical skills room by walking up the stairs. The wearers were also asked to dial a number by calling the Practice Center, lie on the bed and then sit down and get out of bed, sit in a chair and complete a demographic information form. They were also asked to pour water from a pitcher into a glass and drink it, take a few chocolate pills out of the cover and eat them, and finally eat ice cream with a spoon. Only once per student for two hours for two hours, experienced the restrictions of an elderly in this suit. The training in the control group included the usual undergraduate nursing training through participating in the adult-elderly theory classes and its internship.

In the evaluation phase, the knowledge questionnaire about the Palmore facts on aging quiz (FAQI) [22] and the Kogan’s attitudes toward older people scale (KAOPS) [23] were completed by both experimental and control groups before the study and a month after the intervention completion. Therefore, the data collection tool in this study was in the form of collecting demographic information including age, gender, marital status, GPA of the previous semester, and history of taking elderly care courses except for college classes. Part 2: The knowledge questionnaire was FAQI. This questionnaire consists of 25 questions in three dimensions that have the answers of three options “Yes”, “No”, and “I do not know”. The instrument dimensions are: An individual’s knowledge about existing misconceptions about the elderly (10 items), physical condition (5 items), mental condition, and social status of aging (10 items). The number one was given to the correct answer and the number zero was given to the wrong answer and the total score range was from 25 − 0 and a higher score indicates a better situation and a higher level of knowledge and awareness [24]. The validity and reliability of the Persian version of FAQI were confirmed by calculating the Cronbach’s alpha coefficient (0.81) as well as the intra-class correlation of /98 [25].

The Palmore facts on aging quiz available from: https://academic.oup.com/gerontologist/article/20/6/669/629690.

KAOPS has 34 questions and two subscales of appreciation (17 questions) and prejudice (17 questions). The terms of this questionnaire are graded on a seven-point scale 1-Strongly disagree 2-Disagree 3-Somewhat disagree 4-Neither agree nor disagree 5-Somewhat agree 6-Agree 7-Strongly agree. In this questionnaire, the even-numbered questions are scored in reverse. Even-numbered questions measure prejudice towards the elderly and odd-numbered questions measure appreciation towards the elderly. The lowest score is 34 and the highest score is 238. The higher the score, the more positive the attitude towards the elderly [26]. The validity and reliability of the Persian version were confirmed by Cronbach’s alpha for the whole scale of 0.87 [27].

The Kogan’s attitudes toward older people scale available from: http://docplayer.net/15125940-Student-attitudes-about-older-adults-caring-and-cultural-assimilation.html.

### Ethical considerations

 The study was carried out by obtaining permission from the ethics committee of Shahid Sadoughi University of Medical Sciences of Yazd with the ethics ID: IR.SSU.REC1398.170 and informed written consent was obtained from students to participate in the study. The confidentiality of the information and the voluntary initiation and continuation of the cooperation in the study and the lack of influence on scores were described. Also, the experience of wearing aging suits was simulated for students in the control group at the end of the study.

### Data analysis

After collecting data using SPSS version 16 and using mean and standard deviation statistics and independent t-test to compare the mean scores of knowledge and attitude towards aging in the two groups, the paired t-test was performed to compare knowledge and attitude scores at the beginning and end of the intervention and test; the significance level was considered 0.05 in all tests. Adherence to the normal distribution was examined by the Kolmogorov-Smirnov test. The test is performed with a 95 % confidence level, i.e. if the significance level value is less than 0.05; the distribution is not normal. Due to the fact that in this study the p.value were more than 0.05, had normal distribution data.

## Results

There were a total of 84 nursing students of 5th semester of which 43 were in class A and 41 in class B. Indeed, three students in each class were entered because of having a nursing diploma, and four students in class A were excluded because of the history of home elderly care, one person because of unwillingness to participate, 2 students were also excluded from the study in class B because of being a guest in the last semester from another university and one person due to back pain. Therefore, 35 people were selected in the experimental group and 35 people in the control group. In terms of gender, the majority of participants in both groups were female: the experimental group 20 (57.1 %), the control group 19 (54.3 %) and were single: the experimental group 29 (82 %), the control group 25 (72 %) and, by using Chi-square test, had no significant difference (*p* > 0.05). The age mean of the experimental group was (20.85 ± 1.16), and the control group was (21.25 ± 2.20), the average mean of the experimental group was (16.4 ± 1.1), and the control group was (15.4 ± 1.4); the independent t-test showed no significant difference between the age of the two groups (*p* > 0.05) but the average of the two groups had a significant difference (*p* < 0.004). In terms of residence, the majority of the test group were native and lived in a private home 32 (91 %), but the control group were non-native and lived in a student dormitory 28 (80 %) and had a significant difference using the Chi-square test (*p* < 0.001).

Based on the findings in the pre-test in the knowledge components about aging and total knowledge, no significant difference (*p* = 0.1) was found between the experimental group (9.2 ± 2.6) and the control group (10.4 ± 2.9). In the pre-test, no significant difference was found between the two groups about each knowledge component (the knowledge about misconceptions about aging) (*p* = 0.8) and (knowledge of the students’ psycho-social status) (*p* = 0.21) and (the knowledge towards the physical condition of the aging) (*p* = 0.1). But in the post-test in each component, the total score in the experimental group was (15.3 ± 3.5) and in the control group was (11.3 ± 2.6), that a significant difference was reported (*p* = 0.0001). In the experimental group of knowledge components about the elderly (the knowledge about misconceptions about aging (*p* = 0.001) and the knowledge about the psycho-social status of students (*p* = 0.001) and (knowledge about the physical condition of the elderly (*p* = 0.003), a significant difference was reported after the intervention (Table 1).

**Table 1:** The comparison of domains scores and total knowledge of students compared to the elderly before and after the study in the experimental and control groups

Time	Students' knowledge of the elderly	The experimental group		The control group		Independent T-test
		M	SD	M	SD	
**Before the study**	The knowledge of the misconceptions about aging	4.05	1.45	3.97	1.7	*p* = 0.8
	The knowledge towards the physical condition of the aging	2.2	1.1	2.9	1.28	*p* = 0.1
	The knowledge of the psycho-social status of the elderly	3.2	1.3	3.7	1.85	*p* = 0.2
	The total knowledge	9.2	2.6	10.4	2.9	*p* = 0.1
**After the study**	The knowledge of the misconceptions about aging	6.3	1.64	4.8	1.38	*p* = 0.001
	The knowledge towards the physical condition of the aging	3.9	0.91	3.1	1.1	*p* = 0.003
	The knowledge of the psycho-social status of the elderly	6.8	2.4	4	1.89	*p* = 0.001
	The total knowledge	15.3	3.5	11.3	2.6	*p* = 0.0001

The mean score of total students’ attitudes toward the elderly before the intervention in the experimental group was (114.69 ± 8.4) and in the control group was (113.34 ± 13.6). There was no significant difference between the experimental and control groups before the intervention in terms of the total attitude (*p* = 0.6). The prejudice score of the elderly in the experimental group and the control group was (56.6 ± 6.2) and (56.3 ± 7.1), respectively which had no significant difference between the two groups in terms of prejudice towards the elderly before the intervention (*p* = 0.6). The comparison of the appreciation of the experimental group with the control group was (58.06 ± 6.3) and (56.9 ± 10.9), respectively. Indeed, both groups had a significant difference in terms of this area before the intervention (*p* = 0.01). In the post-test of the intervention group, the total score of attitude towards the elderly in the experimental group was (157.3 ± 10.7) and in the control group was (108.5 ± 16.6). After the intervention in the experimental and control groups, a significant difference was found in the areas of attitude towards the elderly (prejudice) (*p* = 0.01) and (appreciation) (*p* = 0.001) and total attitude (*p* = 0.0001) (Table 2).

**Table 2:** The comparison of attitude scores of the domains and total students towards the elderly before and after the study in the experimental and control groups

Time	Students' attitude of the elderly	The experimental group		The control group		Independent T-test
		M	SD	M	SD	
**Pre-test**	Prejudice to the elderly	56.6	6.2	56.3	7.1	***p*** **= 0.6**
	Appreciation of the elderly	58.06	6.3	56.9	10.9	***p*** **= 0.01**
	The total knowledge	114.69	8.4	113.34	13.6	***p*** **= 0.6**
**Post-test**	Prejudice to the elderly	78.5	5.7	52.6	10.1	***p*** **= 0.01**
	Appreciation of the elderly	78.8	6.9	52.9	8.4	***p*** **= 0.001**
	The total knowledge	157.31	10.7	108.5	16.6	***p*** **= 0.0001**

Based on the findings in the experimental group, a significant difference was found in the knowledge areas about aging and the total knowledge in pre-test and post-test (*p* = 0.001). But in the control group, except for the area of knowledge on misconceptions about aging (*p* = 0.001), no significant difference was found in other areas and the total score (*p* < 0.05) (Table 3).

**Table 3:** The comparison of the domain scores and total knowledge of students compared to the elderly in the experimental and control groups before and after the study

Time	Students' knowledge of the elderly	Pre-test		Post-test		Paired t-test
		M	SD	M	SD	
**The experimental group**	The knowledge of the misconceptions about aging	4.05	1.45	6.3	1.64	***p*** **= 0.001**
	The knowledge towards the physical condition of the aging	2.2	1.1	3.9	0.91	***p*** **= 0.001**
	The knowledge of the psycho-social status of the elderly	3.2	1.3	6.8	2.4	***p*** **= 0.001**
	The total knowledge	9.2	2.6	15.3	3.5	***p*** **= 0.001**
**The control group**	The knowledge of the misconceptions about aging	3.97	1.7	4.8	1.38	***p*** **= 0.001**
	The knowledge towards the physical condition of the aging	2.9	1.28	3.1	1.1	***p*** **= 0.9**
	The knowledge of the psycho-social status of the elderly	3.7	1.85	4	1.89	***p*** **= 0.3**
	The total knowledge	10.4	2.2	11.3	2.6	***p*** **= 0.3**

The mean score of the total students’ attitudes toward the elderly and areas in the experimental group in the pre-test and post-test was significantly different (*p* < 0.05). However, in the control group, comparing post-test and pre-test scores, the total score of attitude towards the elderly and area had no significant difference (*p* > 0.05) (Table 4).

**Table 4:** The comparison of the domain scores and total students’ attitudes toward the elderly before and after the study in the experimental and control groups

Time	Students' attitude toward the elderly	Pre-test		Post-test		Paired t-test
		M	SD	M	SD	
**The experimental group**	Prejudice to the elderly	56.6	6.2	78.5	5.7	***p*** **= 0.001**
	Appreciation of the elderly	58.6	6.3	78.8	6.9	***p*** **= 0.001**
	The total knowledge	114.69	8.4	157.31	10.7	***p*** **= 0.0001**
**The control group**	Prejudice to the elderly	56.3	7.1	52.6	10.1	***p*** **= 0.06**
	Appreciation of the elderly	56.9	10.9	52.9	8.4	***p*** **= 0.1**
	The total knowledge	113.34	13.6	108.5	16.6	***p*** **= 0.3**

## Discussion

In the present study, the aging simulation was carried out in a tangible and real way to create the opportunity of the aging experience and its effect on students’ knowledge and attitude toward aging was investigated. Based on the findings, both groups have earned a score of less than half in the pre-test, which indicates poor knowledge and had no significant difference. The present results showed that after the intervention, the average total score of knowledge of the experimental group was higher than half and although the control group has improved slightly compared to before the score intervention, the score is still less than half and the difference between the two groups was significant. In the present study, in the post-test, the intervention group had a significantly higher score in attitude improvement, while the control group had a weak attitude and the difference was significant. Therefore, it seems that simulation has been effective in increasing students’ knowledge by wearing an aging suit.

Specialized knowledge is important in nursing care. Ravanipour et al. (2012) stated that the majority of nurses had average knowledge about aging and none of them acquired high knowledge and there is a need to take measures to improve knowledge [28]. Forouzandeh (2018) also argues that the amount of knowledge of the facts on aging is relatively low among caregivers [29]. Pakpour (2020) states that university educational programs and the provided in-service programs to nurses have not been enriched and have failed to reach the desired level of students and nurses’ knowledge about the facts on aging [30].

The present results showed that after the intervention, the average total score of knowledge of the experimental group was more than half and although the control group has improved slightly compared to before the score intervention, the score was still less than half and the difference between the two groups was significant. Other findings showed that both experimental and control groups have acquired score less than half in attitudes before the study, which indicates the students’ poor attitude towards the elderly and had no significant difference. In this regard, Hweidi (2006) showed that effort to improve the attitude of nursing students towards the elderly is necessary [31]. In the present study, in the post-test, the intervention group has acquired a significantly higher score and had attitude improvement, while the control group had a weak attitude and the difference was significant. In this regard, Chen et al. (2015) showed that the attitude and empathy of nursing and pharmacy students had improved significantly after participating in the aging simulation program [7, 15]. Adib Haj Bagheri et al. (2014) write that a one-day walk in a nursing home was associated with improving the attitudes toward the elderly and that care conditions for the elderly may have influenced how attitudes change [32]. Torkshavand et al. (2020) showed that the learning group based on simulation had greater and more lasting improvements in increasing the knowledge and skills of the older patients than the lecture-based group [33]. Similar to the present study, Sari et al. (2020) used an aging suit as a real simulation which showed that the mean scores of the KAOPS has statistically significantly increased after wearing an aging suit [9].

The results showed that the attitude has improved in the experimental group after the intervention and a significant difference was found, while in the control group before and after the intervention, no difference was found in attitude. Therefore, it seems that wearing the aging suit has been associated with creating a positive attitude towards aging. Similar to the present study, Da Nova et al. (2019) investigated the effect of the aging simulation game on nursing students’ attitudes toward the elderly which the attitudes toward the elderly were significantly improved by the KAOPS after the intervention [8]. Braude et al. (2015) also wrote in the evaluation of an aging simulation training program that quantitative analysis of the questionnaire before and after the period showed a significant improvement of the reported confidence in the management of aging scenarios [10].

One of the limitations of the present study was two hours of wearing suit due to the many programs of students, which is suggested to be used in the future research for a longer period and to design and implement more daily activities that the elderly face and the persistence of its effectiveness at longer times will be investigated. Another limitation was that the students had taken adult-senior theory and internship courses, but it was identical in both groups.

## Conclusions

Findings of the present study in relation to the use of aging restrictions simulation showed that wearing elderly suit has led to a better understanding of nursing care and the knowledge and attitude of nursing students had significantly improved after the intervention. Therefore, it seems that due to the increase in the elderly population and the necessity for the existence of positive knowledge and attitude in health care providers, simulation can be used as easy and simple access, as an effective educational method to improve the knowledge and attitude of medical students by wearing the aging suit; also the clinical skills rooms can easily be equipped with the necessary facilities to simulate the elderly, especially at very low costs. There is also a need for further studies to develop methods to better simulate the restrictions of aging. Also, it is suggested that depending on the grade of nursing students, clinical practice, and in accordance with the nursing curriculum, wear the age simulation suit and its effect be investigated with different study grades.

## Data Availability

The datasets used and/or analyzed during the current study are available from the corresponding author (KHN) on reasonable request.

## Abbreviations

FAQI: Palmore facts on aging quiz
KAOPS: Kogan’s Attitudes toward Older People scale
M: Mean
SD: Standard Deviation

## References

[1] Morley JE (2020). The Future of Geriatrics. J Nutr Health Aging.

[2] Mirzaie M, Darabi S (2017). Population Aging in Iran and Rising Health Care Costs. Salmand Iran J Ageing.

[3] Moran S, Chen Y, Ruthie A, Nir Y (2007). Alterations in IGF-I affect elderly: role of physical activity. Eur Rev Aging Phys Act.

[4] Ravenswood K, Douglas J, Haar J (2017). Physical and verbal abuse, work demands, training and job satisfaction amongst aged-care employees in the home and community sector. Labour Ind J Soc Econ Relat Work.

[5] Zhao RC, Stambler I (2020). The urgent need for international action for anti-aging and disease prevention. Aging Dis.

[6] Kim YK, Kwon S (2017). Effects of empathy and attitude in caring for elders by nurses in geriatric nursing practice in long-term care hospitals. J Korean Gerontol Nurs.

[7] Chen AM, Kiersma ME, Yehle KS, Plake KS (2015). Impact of the Geriatric Medication Game® on nursing students’ empathy and attitudes toward older adults. Nurse Educ Today.

[8] Silvia Fernandes C, Couto G, Afonso A. An aging simulation game's impact on the attitudes of nursing students. Nursing Practice Today. 2019;6(3):142-51.

[9] Sari D, Taskiran N, Baysal E, Acar E, Cevik Akyil R (2020). Effect of an aged simulation suit on nursing students’ attitudes and empathy. Eur Geriatr Med.

[10] Braude P, Reedy G, Dasgupta D, Dimmock V, Jaye P, Birns J (2015). Evaluation of a simulation training programme for geriatric medicine. Age Ageing.

[11] Vanlaere L, Coucke T, Gastmans C (2010). Experiential learning of empathy in a care-ethics lab. Nurs Ethics.

[12] Gilligan AM, Loui JA, Mezdo A, Patel N, Lee JK. A Comparison of Pharmacy Students’ and Active Older Adults' Perceptions Regarding Geriatric Quality of Life. Am J Pharm Educ. 2014;78(1):1-8. 10.5688/ajpe78110 PMC3930234 24558278

[13] Tremayne P, Burdett J, Utecht C. Simulation suit aids tailored care. Nurs Older People. 2011;23(7):19-22. 10.7748/nop2011.09.23.7.19.c8678 21980792

[14] Tjoflåt I, Våga BB, Søreide E (2017). Implementing simulation in a nursing education programme: a case report from Tanzania. Adv Simul.

[15] Chen AM, Kiersma ME, Yehle KS, Plake KS. Impact of an aging simulation game on pharmacy students’ empathy for older adults. Am J Pharm Educ. 2015;79(5):1-10. 10.5688/ajpe79565 PMC4571055 26396274

[16] Plakiotis C (2016). Clinical simulation training in geriatric medicine: A review of the evidence and lessons for training in psychiatry of old age. GeNeDis.

[17] Eost-Telling C, Kingston P, Taylor L, Emmerson L (2021). Ageing simulation in health and social care education: A mixed methods systematic review. J Adv Nurs.

[18] Muroya K, Sato H, Deguchi Y, Takeyama Y, Shono I, Kanayama M (2004). Evaluation of students’ learning through simulation experience study in gerontological nursing education–understanding the elderly person and the role of the caregiver. J UOEH.

[19] Pazargadi M, Sadeghi R (2011). Simulation in nursing education. Bimonthly Educ Strateg Med Sci.

[20] Kane J, Pye S, Jones A (2011). Effectiveness of a simulation-based educational program in a pediatric cardiac intensive care unit. J Pediatr Nurs.

[21] Adib-Hajbaghery M, Mohamadghasabi M, Masoodi alavi N (2014). Effect of an Elderly Care Program on the Nursing Students’ Attitudes Toward the Elderly. Salmand Iran J Ageing.

[22] Palmore E (1980). The facts on aging quiz: a review of findings. Gerontologist.

[23] Kogan N (1961). Attitudes toward old people: The development of a scale and an examination of correlates. J Abnorm Soc Psychol.

[24] Cowan DT, Fitzpatrick JM, Roberts JD, While AE (2004). Measuring the knowledge and attitudes of health care staff toward older people: Sensitivity of measurement instruments. Educ Gerontol.

[25] Nahid Rejeh Majideh, Heravi-Karimooi Ali, Montazeri Mahshid (2012). Psychometric properties of the Iranian version of the Facts on Aging Quiz (FAQI). Health Monit J Iran Inst Health Sci Res.

[26] Yen C-H, Liao W-C, Chen Y-R, Kao M-C, Lee M-C, Wang C-C (2009). A Chinese version of Kogan’s attitude toward older people scale: reliability and validity assessment. Int J Nurs Stud.

[27] Rejeh N, Heravi-Karimooi M, Foroughan M, Nikkhah M, Azam B (2016). The Persian version of Attitudes to Ageing Questionnaire (AAQ): a validation study. Payesh (Health Monitor).

[28] Ravanipour M, Dadaeen A, Jahanpour F (2015). Measuring nurses\‘knowledge about the facts of aging period in educational hospitals of Bushehr in 2012. Iran J Geriatr Nurs.

[29] Frouzandeh S, Foroughan M, Hosseini MA, Farhadi A, Biglarian A (2018). The relationship of nursing home caregivers’ awareness and attitude towards elderlies with their job. J North Khorasan Univ Med Sci.

[30] Packpour V, Allahverdi Mamaghani R, Kianian T (2020). Comparison of Knowledge and attitude of Nursing Students and Nurses towards facts of aging period in 2019. J Gerontol.

[31] Hweidi IM, Al-Obeisat SM (2006). Jordanian nursing students’ attitudes toward the elderly. Nurse Educ Today.

[32] Adib-Hajbaghery M, Mohamadghasabi M (2014). Effect of an elderly care program on the nursing students’ attitudes toward the elderly. Iran J Ageing.

[33] Torkshavand G, Khatiban M, Soltanian AR (2020). Simulation-based learning to enhance students’ knowledge and skills in educating older patients. Nurse Educ Pract.

